# Provision of a Medicines Information Service to Consumers on Facebook: An Australian Case Study

**DOI:** 10.2196/jmir.4161

**Published:** 2015-11-23

**Authors:** Arcelio Benetoli, Timothy F Chen, Sarah Spagnardi, Troy Beer, Parisa Aslani

**Affiliations:** ^1^ Faculty of Pharmacy The University of Sydney Sydney Australia; ^2^ NPS MedicineWise Sydney Australia

**Keywords:** medicines information, Facebook, pharmacists, consumers, information services, drug information services, social media

## Abstract

**Background:**

Social networking sites (SNSs) have changed the way people communicate. They may also change the way people seek health advice.

**Objective:**

This study describes the provision of a medicines information service on Facebook to individual consumers. It aimed to discuss the pros and cons, and inform health and pharmacy stakeholders and researchers about the opportunities and challenges of providing such a service.

**Methods:**

We adopted an exploratory approach using a case study method.

**Results:**

NPS MedicineWise, an independent, not-for-profit Australian organization, runs a public question-and-answer service on Facebook, dubbed Pharmacist Hour. Consumers following the organization’s Facebook page are invited to post medication-related questions often with a suggested health topic. A wide range of questions and comments are posted related to medication usage. The pharmacist answers the queries, providing evidence-based medicines information and using consumer-friendly language, during the specific 1-hour period. The most popular questions in the past 12 months were related to adverse effects, treatment options for conditions, and drug interactions. The service had a mean number of engagements (defined as a like or share of the Pharmacy Hour post) of 38 (SD 19) people and a mean 5 (SD 3) questions per session.

**Conclusions:**

The Pharmacist Hour Facebook service addresses the medicines information needs of consumers and indirectly promotes other appropriate and relevant NPS MedicineWise products and services to further assist consumers. The service offers a new medium for a quality use of medicines organization committed to promoting awareness about the correct and safe use of medicines in Australia.

## Introduction

Social networking sites (SNSs) are widely used and have changed the way people communicate. They are defined as Web-based services that allow individuals to create a profile within a bounded system, articulate that profile to a list of other users, and view and traverse that list and those made by others within that system creating a network [1]. There is a plethora of SNSs, but Facebook, founded in 2004, is the most popular with 1.35 billion monthly active users (or 864 million daily active users) as of September 30, 2014 [2]. The chief reasons for using SNSs are to connect with other people, keep in touch and make plans with friends, locate old friends, learn about events, share photos, pass time, entertainment, and information gathering [3-6].

With all its functionalities and capabilities, it is not surprising that SNSs are used for health-related purposes. Consumers, particularly younger people with chronic diseases, are grouping themselves in Facebook to share and compare experiences with the disease and treatment in a supportive and readily accessible environment [7]. As the demographics of Facebook shifts to include more of those aged 50 and older [8], an increase in health-related activities might follow suit. It is expected that social media can help the delivery of patient-centered, high-quality, and affordable health care in the future [9].

Social media has changed the way the general public accesses health information [10] and it might represent an additional option consumers have to communicate with their providers while also enabling the providers to offer care to those who cannot or prefer not to come to them [11]. The provision of services to consumers using SNSs has potential to provide an efficient, consumer-friendly, and cost-effective channel for health care systems. It is believed that a Facebook service trial for pediatric medical consultations in Finland reduced the need for further contacts with a health care service in more than half of cases [12]. Additionally, a number of indicators, such as research papers in the field, demonstrate that the use of social media in the health care context is growing [13].

As the pharmacists’ role in health care evolves, the way they communicate with their clients may change depending on the technologies available. Shcherbakova and Shepherd [14] showed in a statewide study in the United States that pharmacists providing medication therapy management services at their workplace are more likely to communicate with patients via text, email, or social media. Although SNSs could be used to provide pharmaceutical services to consumers, no research has been conducted so far to address the feasibility of this kind of service. Therefore, this study aimed to provide a detailed description and modus operandi of a public medicines information service provided to individual consumers through Facebook by a national not-for-profit Australian organization. It also aimed to discuss the pros and cons of the service and inform health and pharmacy stakeholders and researchers about the opportunities and challenges of providing a similar service.

## Methods

This exploratory study used a case study format. A case study is defined as an in-depth multifaceted investigation of a single social phenomenon using qualitative research methods [15]. It provides a holistic in-depth approach, which is particularly valuable in the generation of new ideas and theories in social science [15]. This qualitative methodology is of great value in health care because it can be used to inform professional practice or evidence-informed decision making in both clinical and policy realms [16].

The case in this paper does not represent a family or group of cases; instead, it was chosen because of its uniqueness. To our knowledge, it is the first time a public medicines information service provided to individual consumers on a social networking site, namely Facebook, is described and discussed in a peer-reviewed journal. The data presented here were collected through an in-depth semistructured interview with the pharmacist in charge of the Facebook service and direct retrospective observation of the service during 12 months. The interview was conducted as part of a broader research study, which aimed to investigate the role and impact of social networks in the delivery of health care by pharmacists. The interview guide included pharmacists’ understanding of social media, its use as part of their professional practice, and their perceptions of its use in health care and in the pharmacy profession by both pharmacists and consumers. During the interviews with one of the participants (SS), the researchers learned about the Facebook service as an example of the use of social media by pharmacists as part of their professional practice. The authors chose to specifically present the findings of the interview pertaining to the NPS MedicineWise Facebook service as well as further information about this service. This study received ethical approval from the Human Research Ethics Committee of the University of Sydney.

## Results

### The Organization: NPS MedicineWise

NPS MedicineWise, known as the National Prescribing Service prior to 2009, is an independent and not-for-profit organization committed to quality use of medicines, specifically improving the way health technologies, including medicines and medical tests, are prescribed and used in Australia. NPS MedicineWise provides evidence-based educational programs for general practitioners, pharmacists, nurses, and students. The educational activities are delivered online, face-to-face, or as group discussions. Participants are able to obtain continuing professional development points for participating in the activities. The activities include case studies, clinical e-audits, online courses, educational visits, and pharmacy practice reviews.

Since 2003, NPS MedicineWise has been committed to promoting discussions about basic medicine-related issues in the community via consumer education programs and conducting health campaigns, such as Be Medicinewise Week [17]. NPS MedicineWise has a website [18] where both health care professionals and consumers can access NPS MedicineWise materials for continuing education and self-management, respectively. Another service directed to consumers is Medicines Line [19], which is a telephone service aimed at providing information on prescription, over-the-counter, and complementary and alternative medicines. The Medicines Line Services team is staffed by registered pharmacists, employed by NPS MedicineWise, who have been trained to deliver medicines information to consumers. At any time, 3 to 5 pharmacists are on roster to manage incoming telephone calls. Medicines Line operates in collaboration with Healthdirect Australia in all Australian states and territories (except Queensland and Victoria) and promotes quality use of medicines and provides information that is independent, evidence-based, appropriate and safe, and encourages responsible use of medicines by increasing public awareness about medicines. Specific information provided includes how a medicine works, how to take medicines, adverse effects and interactions with other medicines, medicines during pregnancy and breastfeeding, medicines for children, storage of medicines, how to obtain consumer medicine information for prescription medicines, and referrals to reliable services and support organizations. Medicines Line does not provide emergency services, medical advice, or second opinions on the appropriateness of medicines recommended by health care professionals. NPS MedicineWise also provides consumers with a free smartphone app, called MedicineList+, with features such as reminders of how and when to take medicines, calendar alerts for refills, a barcode scanner to input medicines, and the ability to record details about medical test results which are displayed graphically.

### NPS MedicineWise and Social Media Presence

Social media represents a new opportunity to engage with consumers. NPS MedicineWise set up a social media team consisting of 2 staff: the social media coordinator and the manager of digital services / digital communications advisor. In 2011, it launched a Facebook page to target key demographics in the Australian population with health information. The organization’s social media presence aims to “provide information to help people with their decisions about managing health, medicines, and medical tests” [20]. As of November 15, 2014, the NPS MedicineWise Facebook page had 45,700 followers (people who liked the page and see its posts on their newsfeed). The demographic breakdown was more than 85% women with the majority in the 25 to 54 years age bracket.

#### The Pharmacist Hour Facebook Service

The initial aim of the Facebook page was to engage consumers through weekly topics. However, due to social media interactivity, consumers started to post medicine-specific questions related to their own needs. To fulfill the consumers’ need for a more interactive presence on Facebook, NPS MedicineWise designed a free-of-charge public service called Pharmacist Hour. Followers of NPS MedicineWise’s Facebook page were invited to post medicine-related questions, with a focus on a specific health topic suggested by the organization. Pharmacists from the Medicines Line team answered the Facebook questions, which were then posted online during the specified 1-hour period each week.

This new service posed new challenges to the clinical team; namely, a change in the mode of information transmission (ie, from verbal to written) and audience reach (ie, from one-to-one to unlimited viewers):

We were all probably quite hesitant about it [Pharmacist Hour service] because we’re used to dealing with so much one-on-one with people over the telephone, people were concerned about their writing skills. So they’re used to talking, not putting information into a written form, so that was interesting...they were very concerned about the ramifications of the work...concerned about how much information can you give to someone on Facebook because it’s not, even though you’re answering a question, that question and answer is seen potentially by thousands of people.

Initially, when the service was implemented, formal staff training was not conducted. Any issue that arose was addressed through extensive team discussions until the team was completely comfortable in delivering the service and over time the service became part of the team’s routine activities:

[At the beginning] we would have big discussions before sending the answer to the social media team, you know...it’s been interesting, but over the past 9 months I’d say it’s become much more comfortable and it’s sort of embedded now, as part of our weekly process.

Pharmacist Hour is considered an appropriate tool for consumer engagement on Facebook for a number of reasons. Firstly, it focuses medicines-related questions into a specific time period allowing the service to be responsive, but reduces the need to schedule Medicines Line staff outside this time period. Secondly, it is a service provided for people who follow the Facebook page. Thirdly, it provides an opportunity for NPS MedicineWise to partner with other health-focused organizations (eg, Asthma Australia and the Stroke Foundation) on specific health issues.

Generally, consumers posted questions before the specified time and the social media team posted a standard reply stating that the pharmacist would reply back during the Pharmacist Hour. Questions posted after the specified Pharmacist Hour were also addressed, but not with the same immediacy. The promotion of the Pharmacist Hour service reduced the frequency of nonscheduled questioning.

**Figure 1:**
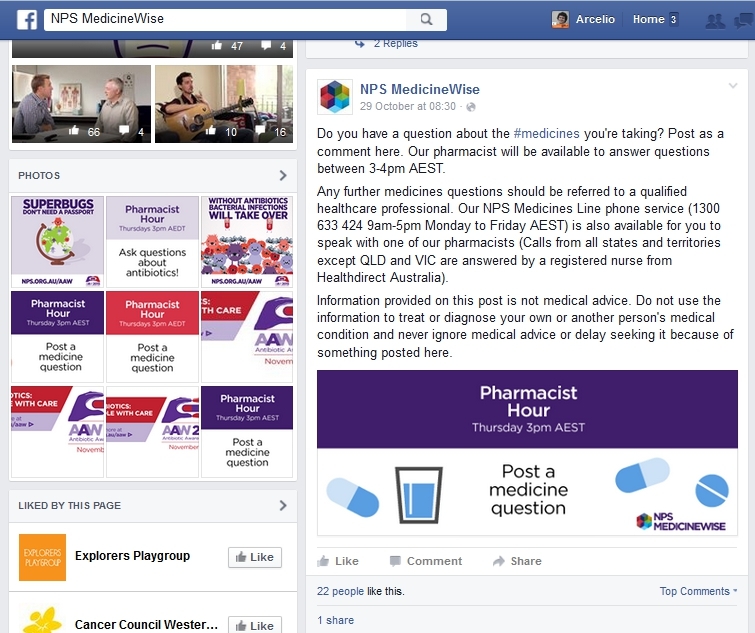
Screenshot of NPS MedicineWise Facebook advertising the Pharmacist Hour service.

This weekly Facebook service first started in October 2013 and currently runs on Thursdays from 3 pm to 4 pm Australian Eastern Standard Time. Figure 1 shows an example of an NPS MedicineWise invitation post for the Facebook service. The direct public Facebook service complements the NPS MedicineWise social media presence promoting the safe use of medicines by the provision of evidence-based information. Additionally, this service provides insight into the types of questions posted and the type of language used by people to describe their issues online. The service also acts as an indirect advertisement about other NPS MedicineWise products and services that may be relevant and appropriate for the people posting medicine-related questions.

#### Engagement With the Facebook Service and Types of Questions Posted

Data gathered from the Facebook page Insight [21], a tool that provides information about the page performance, revealed the engagement and reach of the Pharmacist Hour service (Table 1).

**Table 1:** Data obtained from Facebook Insight for the Pharmacist Hour posts during the first 12 months (October 18, 2013-October 30, 2014).

Facebook page Insight (first 12 months)	Mean (SD) or n
Pharmacist Hour post reach per week, mean (SD)	3776 (1595)
People engaged (shares and likes) with the Pharmacist Hour per week, mean (SD)	38 (19)
Number of people sharing the Pharmacist Hour post per week, mean (SD)	6 (7)
Number of questions per week, mean (SD)	5 (3)
Total number of questions, n	226

Over the first 12 months (October 2013-October 2014), the Pharmacist Hour had a mean reach of 3776 (SD 1595), indicating that 3776 different Facebook users saw the Pharmacist Hour posts in their newsfeed. In terms of engagement [22] (ie, number of unique people who liked or shared the Pharmacist Hour posts), the weekly mean was 38 (SD 19) Facebook users. This included a mean 6 (SD 7; median 3, IQR 1-7) people sharing the Pharmacists Hour post every week.

**Figure 2:**
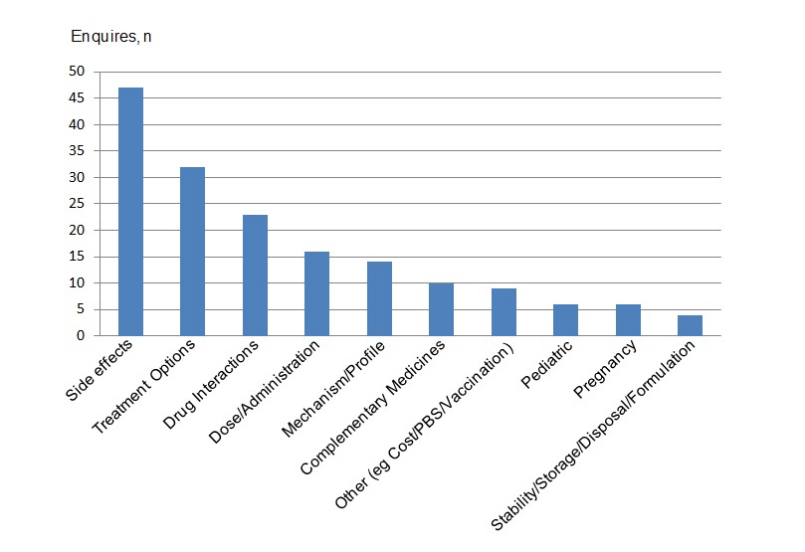
Question categories in the Pharmacist Hour Facebook service in the first 12 months (October 18, 2013-October 30, 2014).

During this 12-month period, a total of 226 questions were posted with a mean number of 5 (SD 3) questions per week. Figure 2 details the prevalence of questions by topic according to the classification used by the organization for recording purposes. A total of 27 questions were found to contain content that belonged to more than one category. The categorization of the questions asked was conducted by NPS MedicineWise based on their internal procedures. Multiple categorization of Facebook posts and other enquiries is used to ensure accurate categorization and maximal information retrieval when searching for posts. The most common topic was adverse effects of medicines followed by questions related to treatment options for conditions, drug interactions, and dose and administration.

### Response Process

The social media team responsible for managing the Facebook page content was in charge of collecting the questions and posting the answers. As soon as a query was posted, a designated social media staff sent it privately to a designated pharmacist through corporate instant messaging systems or corporate email.

**Figure 3:**
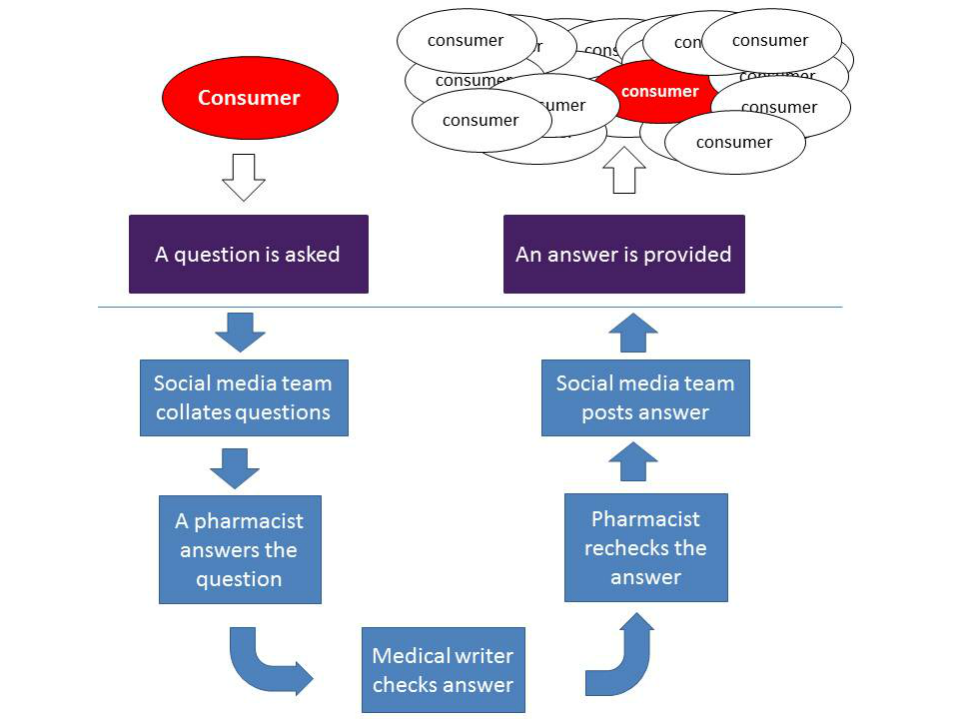
Flowchart of the Pharmacist Hour Facebook service.

The reply followed a NPS MedicineWise–designed procedure. A flowchart of the service is provided in Figure 3. After the answer was formulated by a pharmacist, it was sent to a medical writer and social media staff for editing. This step aimed to reduce the amount of jargon and technical language to make all posts consumer-friendly and easy to understand, plus link to appropriate resources on the NPS MedicineWise website or other resources. The reply was checked again by the pharmacist to certify the clinical content was not changed. Once the answer was approved, the pharmacist sent it with the corresponding question to the social media team for posting. Even though consumers sometimes would post queries before the specified time, all answers were posted during the stated Pharmacist Hour.

All answers followed the NPS MedicineWise protocol and procedures. This ensured that no medical advice or second opinions were given. Moreover, the replies were worded carefully to avoid any misinterpretation. The service followed a question-and-answer format and NPS MedicineWise did not engage in online discussions. There was no information gathering to clarify certain aspects when providing an answer; all replies were made with the information provided by the consumer.

### Concerns and Limitations of the Facebook Service

The pharmacists at NPS MedicineWise were mindful that potentially thousands of people could see the questions and their respective answers through the Facebook service. Indeed, the first year of the service obtained a mean reach of 3776 unique viewers per week.

Because the Facebook page can serve as an open forum, any Facebook user can post answers or comments to the questions. This happened on rare occasions, in which technically correct answers were posted by other followers. To avoid confusion by less Facebook-savvy users, a disclaimer stating that NPS MedicineWise does not endorse anyone else’s replies and that all answers are posted under its name only is included on the Facebook page and on each Pharmacy Hour post.

Another safety measure is that the pharmacist answering the questions is sent only one question at a time and does not see the actual Facebook posts on the live Facebook page. This strategy allows the pharmacist to focus on the question and avoid distraction by reading subsequent questions or comments. As previously mentioned, the collection of questions and posting of answers is the responsibility of the social media team.

Due to consumer safety concerns, the Pharmacist Hour Facebook service was more limited in the depth of information provided compared to the information delivered directly to individual consumers over the telephone. The Pharmacist Hour answers are succinct and straightforward to avoid misinterpretations by both the person who posted the question and other readers. Another limitation is that because a dialog is not developed, it is not possible to gauge consumers’ understanding. In cases in which further information is required to provide an answer, the consumer is advised to contact the Medicines Line service:

In order to provide you with the safest and most appropriate information for your question, we need to gather further information about you and your medicines. By calling NPS Medicines Line on 1300 633 424, we will be able to do this in a much more efficient way.

Whenever appropriate, consumers were simply advised to see their doctor or pharmacist. This procedure also discouraged consumers from sharing private information online, further safeguarding their privacy.

Similarly, questions with few details prevented an answer altogether. For example, if the medication names were needed to answer a specific question, the consumer was advised to call the Medicines Line service. These occasions were seen as an additional opportunity to promote the flagship (telephone) service, Medicines Line.

All Facebook posts were researched, completed, and documented in the Medicines Line database in-line with all enquiries made to the Medicines Line service. They were included in the internal quality assurance activities undertaken by the Medicines Line team regularly. Additionally, all email correspondence between pharmacists and the social media team as well as the responses to the questions posted were recorded and saved.

## Discussion

Although SNSs have the potential to increase communication between pharmacists and consumers, the literature on the professional use of social media by pharmacists is scarce with no mention of provision of clinical services [23]. Delivery of health and medicines information services through Facebook is in its infancy and one approach to developing appropriate and effective services through this popular SNS is to learn about the existing health and medicines information services being delivered, including the strengths and limitations of the approaches used. Therefore, we present a case study about the provision of a direct service to the public by an organization focused on quality use of medicines using Facebook as the medium. We believe that this case study can provide useful insights in the debate about using social networks as a medium in the overall care of patients by pharmacists.

Since the conception of pharmaceutical care in the 1990s [24], there have been huge efforts in moving the pharmacy profession from a product orientation to a patient/consumer orientation. The services that pharmacists offer are no longer restricted to supply of medicines, but also include the delivery of cognitive pharmaceutical services, which are patient-centered and aim to improve patient outcomes. This paradigm shift is being continuously implemented in varying degrees in the pharmacy profession globally. Digital technologies, such as social media, provide additional opportunities for the delivery of cognitive pharmaceutical services. Pharmacists, as experts on medicines, are well equipped and should be encouraged to contribute to these new online tasks.

This study has shown that SNSs, specifically Facebook, can be a medium for the delivery of a public medicines information service to individual consumers. The Pharmacist Hour service has been an effective way of engaging the broad community on the topic of medicines information. The Facebook Insight provided useful service metrics revealing its reach and engagement. Although a mean number of 5 questions were posted per week, a much higher number of people, 38, engaged with the service per week by liking or sharing each Pharmacist Hour post during the service’s first year. The mean reach per week was 3776 (ie, the number of people who saw the post, this includes those who engaged with it). This highlights that consumers were both actively posting questions as well as passively receiving information. A particularly important interaction with the Pharmacist Hour in terms of dissemination was sharing of Facebook service posts on consumers’ own Facebook timelines. This activity increased the reach of the service and can effectively drive more followers to both the Pharmacist Hour and the NPS MedicineWise Facebook page and website (and consequently its available resources).

A wide range of topics related to medicine usage were raised by “online consumers.” Adverse effects was at the top of the list. This reflects consumers’ desire to know about the adverse effects of their medicines [25]. A wish to better understand the pharmacological possibilities available to deal with health issues can also be inferred from the data because the second most popular topic was related to treatment options.

Another study that corroborates the idea presented in this case study, that medicines information can be delivered via the Internet to address individual consumer’s needs, was recently reported in the form of a private chat-based telepharmacy service in Denmark, where answers to queries were not made public [26]. The service operated on behalf of a national pharmacy association and provided counseling services to consumers 24 hours a day, 7 days a week. The service was conceived to ensure quality use of medicines purchased online. However, it was found that the majority of enquiries (89.5%) were not directly related to online purchases.

It is possible that technology savvy consumers are increasingly realizing that the Internet in general, and SNSs in particular, can be used for health-related activities, including the delivery of customized medicines information. Moreover, those aged 50 years and older are increasingly joining Facebook [8], which can potentially contribute to an increased demand for online health services.

Consumers resorting to such innovative online services to obtain tailored medicines information may highlight the limited information they are currently receiving. It may also demonstrate their desire to obtain tailored information that addresses their specific needs rather than broad medicines information that may not be useful for them. Moreover, online services provide an opportunity to ask questions anonymously without attending a community pharmacy and at a time convenient to the consumer. Pharmacists should be encouraged to embrace new communication technologies and understand the possibilities of using them to better care for patients and provide tailored information. As health consumers change (ie, the way they communicate and access information), the pharmacy profession has to be open to adapt to fulfill their needs in terms of effective and safe use of medicines. However, pharmacists should be aware of challenges, limitations, disadvantages, and risks of engaging in the provision of a tailored service through these new media.

Although at first glance the Pharmacist Hour could be perceived as a simple and straightforward service, a more in-depth investigation of the service revealed not only the extensive efforts involved in providing a quality and safe service, but all the challenges. The primary challenge is to ensure that the written information provided is clear and understandable by a broader audience. Although formal training was not initially implemented, processes were put in place to ensure that the written advice provided is of high quality and comprehensible.

Patient confidentiality and professional liability were also issues that concerned the clinical team. Consequently, a well-defined policy for the service was implemented. No additional information is gathered online from the consumers posting the questions. Answers are formulated based only on the initial question and information provided by the consumer. This approach safeguards both the consumer and health care professional. The former are spared from providing personal health information in an open forum and the latter from requesting such information and risking exposing the consumer’s personal information to a broad group of people. This policy ensures users’ confidentiality and privacy [27]. If further information is needed to formulate a reply, consumers are advised to access the Medicines Line service directly. The safety of consumers is also a priority. In addition to providing evidence-based information, when appropriate, the consumer is instructed to contact their doctor or pharmacist for additional advice.

Although the Pharmacist Hour service is free-of-charge, remuneration or a source of revenue to support online services is an important consideration for those planning to use SNSs as a medium for health care delivery. For example, if a community pharmacy decides to engage in the delivery of a service similar to the Pharmacist Hour, it would require a pharmacist dedicated to the task, which would mean extra costs for the pharmacy. Previous literature has identified the need for reimbursement for health care professionals delivering care via social media, such as e-consultations [28]. Because users might not be willing to pay for online services, the expansion of service delivered via mobile technologies might require a change in both culture and operations of health care providers who are used to existing reimbursement models for provision of episodic care [29].

### Limitations and Future Studies

The description and discussion of a single case of a public medicines information service on an SNS will not cover all possibilities that this relatively new communication medium affords. However, this methodological approach has allowed us to focus on understanding the dynamics present within a single setting [30]. Specifically it has allowed us to describe and discuss a medicines information service on Facebook and show health and pharmacy stakeholders that SNS is a new medium for the delivery of medicines information services to empower consumers and foster quality use of medicines.

This study did not focus on evaluating the opinions and perceptions of consumers using the Pharmacist Hour Facebook service or on providing a detailed analysis of the questions asked by the consumers. This information would add important insights about consumers’ needs and preferences for health services sought online. This is a topic for future research.

Further studies about the use of social media to deliver health and medicines information services should be encouraged. All services and interventions should be evidence-based and their impact on consumer outcomes evaluated [31]. Consequently, further assessment of outcomes related to quality use of medicines afforded by social media services is needed.

### Conclusions

This study described and discussed the provision of a not-for-profit medicines information public service to individual consumers on Facebook by NPS MedicineWise, an Australian organization focused on promoting quality use of medicines. The Facebook service dubbed Pharmacist Hour has allowed consumers to post a wide range of medicine-related questions, primarily on adverse effects, treatment options for conditions, and drug interactions. This Facebook service is an additional channel that allows NPS MedicineWise to promote the safe use of medicines by the provision of evidence-based information.

An SNS can be a new medium for the provision of individualized health and medicines information services, in which pharmacists might play a prominent and significant role. Pharmacies and other health care providers planning to implement a similar SNS service to individual consumers should be wary of some challenges highlighted in this case study, such as careful service design, patient confidentiality and privacy, professional liability, quality and extent of information, and resources. To better understand the possibilities and challenges of an SNS in the provision of pharmacy services, the description and analyses of other services are needed.

## Abbreviations

SNS: social networking site
